# Comparing telesimulation-based learning and e-learning as remote education delivery methods in pre-hospital practice

**DOI:** 10.29045/14784726.2024.12.9.3.53

**Published:** 2024-12-01

**Authors:** Chloe Scott, Nigel Rees, Suman Mitra

**Affiliations:** Cardiff University; Whipps Cross Hospital ORCID iD: https://orcid.org/0000-0002-1647-6826; Welsh Ambulance Service NHS Trust ORCID iD: https://orcid.org/0000-0001-8799-5335; Betsi Cadwaldr University Health Board

**Keywords:** acute asthma, e-learning, pre-hospital, simulation, telesimulation

## Abstract

**Introduction:**

Pre-hospital practitioners based at rural and geographically spread-out regions often find it difficult to access education and training for continuous professional development. Distance learning can resolve the dilemma of how to provide high-quality education where the number of participants is small and widely scattered. E-learning is an established teaching modality that has been widely used, whereas telesimulation is a novel teaching tool that has been evolving throughout the past decade. This study aimed to evaluate the experience of e-learning compared to telesimulation for pre-hospital practitioners.

**Methods:**

This study was conducted from January to June 2021. Twenty-six pre-hospital responders were randomly allocated to complete either an e-learning module or a telesimulation session based on acute paediatric asthma. Each participant completed a post-session satisfaction questionnaire for quantitative and qualitative analysis. For the telesimulation session, all participants attended remotely, while the faculty were based on site. The e-learning module was accessed by the participants via the internet.

**Results:**

Both modalities were positively received, with participants agreeing that the learning objectives were met. However, telesimulation was rated significantly higher in terms of engagement (p = 0.044) and suitability (p = 0.033). Qualitative feedback highlighted the immersive and realistic nature of telesimulation as key advantages, while e-learning was appreciated for its flow and stimulating questions.

**Conclusion:**

Telesimulation and e-learning can help learners with restricted availability and geographical challenges. Telesimulation allows learners to work as a multi-disciplinary team despite being scattered across a large geographical area, while e-learning gives learners the flexibility to access education at a convenient time.

## Introduction

Medical education provides society with competent, trained and up-to-date practitioners, who prioritise patient care and commit to maintaining and developing their skills throughout a lifelong career (Swanwick, 2013). Medical education can be delivered by a variety of approaches, such as pedagogical (teaching dependent learners), andragogical (teaching self-directed learners) and heutagogical (managing self-managed learners). Didactic lectures have been the most common pedagogical approach to deliver education (Krishna Teja et al., 2021). Among the pedagogical approaches in medical education, several modalities have been used. The dictating factor is often that of cost and facilitator availability, technology required and digital literacy (Mohammadyari & Singh, 2015). The COVID-19 pandemic necessitated the world rapidly developing and implementing technology-based education, and pre-hospital paramedic practitioners were affected by this along with all the other challenges they faced at the time (Sahi et al., 2020).

Online education can be delivered as either synchronous or asynchronous (Azlan et al., 2020). Synchronous learning, such as online lectures and webinars, allows learners and tutors to gather in real time, enabling live interaction between them (Azlan et al., 2020). Conversely, in asynchronous learning instructors prepare educational content in advance and learners access it at their own pace (Azlan et al., 2020).

### Simulation-based training

Simulation-based learning in healthcare is a synchronous learning event using realistic scenarios to replicate clinical environments, allowing practitioners to enhance various skills, from basic assessments to complex procedures, in a safe and controlled setting (So et al., 2019). This method enables learners to develop fundamental technical and non-technical skills, such as decision making, communication and teamwork, to improve patient outcomes without compromising patient safety (Abelsson et al., 2014). However, simulation training poses challenges due to the high cost of infrastructure and the necessity for both learners and trained technicians to be present in the same place.

### Telesimulation and e-learning

Telesimulation was established by a group of surgeons from the University of Toronto to remotely teach the basics of laparoscopic surgery to developing countries (Burckett-St Laurent et al., 2016). Telesimulation uses telecommunication and simulation to provide simulation-based education, training and/or assessment to learners at an off-site location, eliminating the time and distance barriers of traditional simulation training (Garland et al., 2019; McCoy et al., 2017). This method has been used to teach a variety of healthcare professionals, including anaesthetists (Patel et al., 2020), medical students (Yang et al., 2020) and emergency medical service practitioners (McCoy et al., 2019). This training method was beneficial during the onset of the COVID-19 pandemic, which exacerbated the already limited opportunities for in-person training in clinical and academic settings. In response, educators used telesimulation to minimise interruptions in healthcare education (Palancia Esposito & Sullivan, 2020).

E-learning is an asynchronous, self-paced and didactic learning activity and is a popular method of delivering medical education (Lewis et al., 2014). It is an umbrella term frequently used to describe online learning, distance learning and blended learning (Kumar Basak et al., 2018; Lewis et al., 2014). There is often inconsistency in the definition of e-learning; broadly, it is the process of acquiring information through the use of electronic devices and media (Tamm, 2019). E-learning has expanded healthcare educational capability and has made learning resources more available (Lewis et al., 2014). It allows learners to learn at their own pace (Azlan et al., 2020) and allows information to be disseminated easily so that learners acquire good theoretical knowledge (Azlan et al., 2020; Hugenholtz et al., 2008; Ruiz et al., 2006).

Although medical education has advanced over the decades, allowing distance learning and removing barriers to education, there is limited literature comparing the different pedagogical approaches to distance education.

### Aims and objectives

The aim of this study was to evaluate the experience of e-learning compared to telesimulation. The objectives were to create educational content for telesimulation and e-learning for paramedics and emergency medical technicians from the Welsh Ambulance Service Trust (WAST), to deliver the content via a telesimulation session from a remote facility and an e-learning module and, finally, to evaluate these innovative ways of engaging pre-hospital practitioners as an alternative to face-to-face education and training. Although the preparation, facilitation and delivery, as well as the learner experience, are different in these two pedagogical modalities, the similarity lies in the learning objectives achieved and the accessibility of knowledge dissemination (Nelsen et al., 2020).

## Methods

### Study design and setting

We performed an observational study to evaluate a pre-hospital clinician’s experience of completing an e-learning module compared to a telesimulation-based session. The study was conducted between January and June 2021 in the UK and involved creating and delivering educational content to paramedics and emergency medical technicians from the WAST, which consists of over 3500 members of staff, with responders covering over 20,000 km^2^, ranging from busy city centres to remote rural locations (Welsh Ambulance Services University NHS Trust, n.d.).

### Selection and randomisation

The study was open to all qualified paramedics, advanced paramedic practitioners and emergency medical technicians from the WAST. Participants were recruited through poster and social media advertisement. A total of 26 participants volunteered for this study. Each participant was then randomly allocated to either the e-learning or simulation-based session on a 1:1 ratio.

### Development of teaching and learning materials

Both teaching methods covered the topic of an acute paediatric asthma scenario. The educational content for both sessions was designed to complement prior knowledge and build upon previous clinical experience. The content followed the Joint Royal Colleges Ambulance Liaison Committee (JRCALC) Clinical Guidelines 2019, the national clinical practice guidelines for NHS paramedics (JRCALC, 2019). The learning outcomes were also formulated to be achieved by both teaching methods and are demonstrated below:

1. List differentials for acute breathlessness in a child.
2. Recognise the features of acute asthma in a child using an ABCDE approach.
3. Describe the features of mild, moderate, acute, severe and life-threatening asthma.
4. Identify when adrenaline should be used in acute asthma and state how it should be administered.
5. Formulate a management plan for a child presenting with acute asthma following the JRCALC guidelines.

### Telesimulation design

The simulation-based sessions were delivered using the telecommunication platform Microsoft Teams, software designed to host online meetings allowing real-time broadcasting and screen sharing (https://www.microsoft.com/en-gb/microsoft-teams/group-chat-software). Participants were scheduled to attend one session remotely for a one-hour period. A total of five sessions were delivered over five days. There were between two and four paramedics participating in each session, and they worked as a team during the live simulation. The session content was delivered by trained and experienced faculty based at a facility in North Wales. The session involved a short lecture, pre-brief, simulation and debrief, followed by questions and feedback.

**Figure 1:**
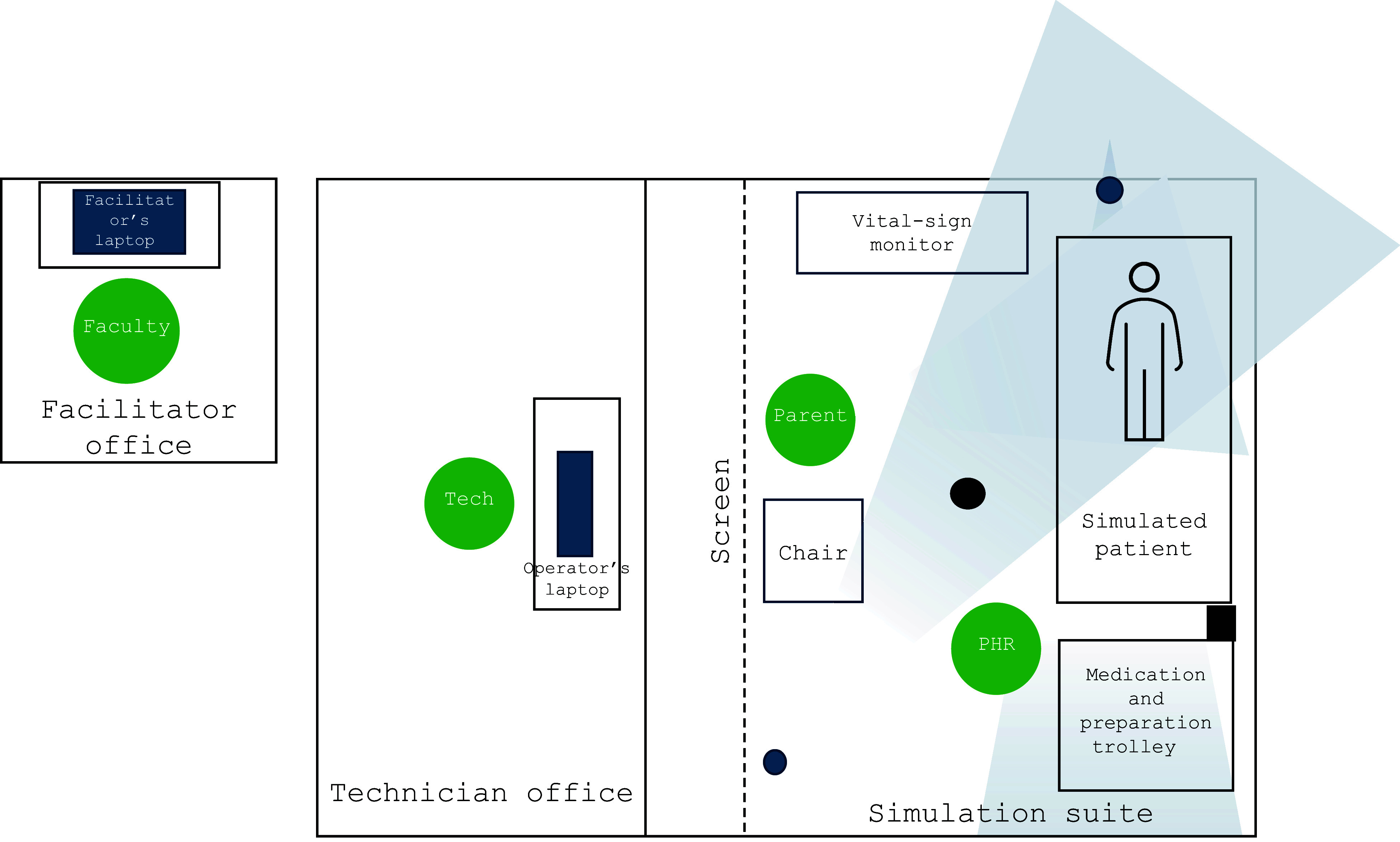
Figure 1. Schematic diagram of the simulation suite, representing the inside of an ambulance. Tech = technician; Parent = facilitator playing the role of a parent (wearing Microsoft HoloLens 2 glasses displaying the view area); PHR = facilitator playing the role of the pre-hospital responder; Faculty = facilitator located in a separate room, leading the session; blue circle = HD PTZ camera located on the ceiling, displaying the view area; black circle = microphone located on the ceiling, feeding into CAE Learning Space™; black rectangle = speaker projecting the voices of the participants into the simulation suite.

**Figure 2:**
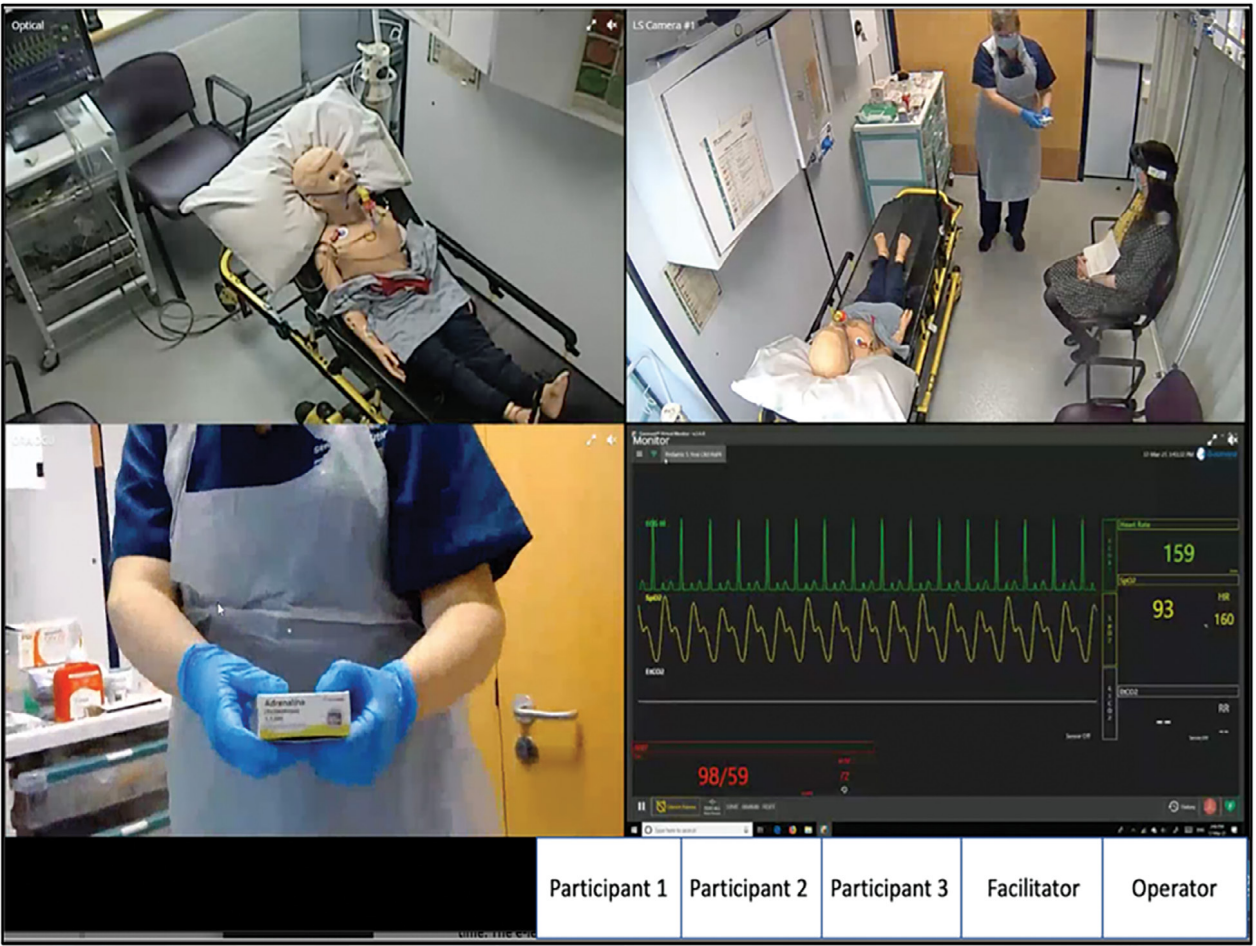
Figure 2. Participants’ view during the simulated scenario on Microsoft Teams. Square boxes represent participants (camera and microphone on), facilitator (camera off, microphone on) and operator (camera off and microphone muted, screen-sharing the feed from CAE Learning Space™ to participants and feeding participant voices through to the speaker located in the simulation suite).

The simulation-based session involved a 7-year-old boy presenting with acute asthma. The scenario started in the back of an ambulance after taking handover from a responder already on scene. A facilitator acted the role of the parent, and a paediatric manikin was used as the patient. Cameras, Microsoft HoloLens 2 smart glasses (https://www.microsoft.com/en-gb/hololens) and an audio microphone were set up to capture the manikin, vital signs monitor, surrounding environment and the voices of the parent and facilitator (Figure 1). This was streamed through the operator’s laptop via CAE Learning Space™, which was screen-shared to the participants through Microsoft Teams (Figure 2). A speaker located in the simulation suite projected the voices of the participants for the facilitators to hear.

The participants were asked to work as a team and to respond to the scenario as they would in their role. They were encouraged to verbalise the actions they would carry out. The facilitator only performed the actions once directed to. The technician reacted to the actions by altering the vital signs displayed on the screen. Another facilitator acted as the parent and responded to the participants; this facilitator also wore the Microsoft HoloLens 2 glasses to provide a first-person perspective of the scenario.

The debrief session was held straight after the scenario, while all participants were online. This was to ensure the learning outcomes were met. The facilitator, who is also one of the authors, kept the discussion strictly to learning objectives and clinical management, being aware that any reflection on the experience may influence the study outcome.

### E-learning design

The e-learning module was delivered using the platform Xerte (https://xerte.org.uk), a tool used to create interactive content for users to access via the internet. The participants were provided with the website link for the e-learning content and were able to complete it in their own time. The e-learning module included a mixture of interactive activities, written information, videos and a short assessment. The e-learning module was designed to take approximately one hour to complete.

### Data collection

Data were collected by the use of questionnaires. All participants completed the same pre- and post-session questionnaire. Demographic data were collected via pre-session questionnaires to compare the skill set and experience of participants in both the telesimulation and e-learning groups. The post-session questionnaire contained a five-point Likert scale to gather more information regarding the delivery of the teaching sessions. There was also the opportunity for the participants to provide qualitative written feedback, recall learning points and comment if the training session would affect their future practice.

### Outcomes

The primary outcome of this comparative study was to determine the experience of telesimulation compared to e-learning. Secondary outcomes included identifying key learning points and how this training would affect participants’ future practice.

### Data analysis

For the Likert-style responses, data were summarised into those who agree or disagree / were neutral; a chi-squared test was used to compare variables. Significance level was set at p <0.05. For the qualitative data, repeated words, sentences or ideas were grouped into themes and a thematic analysis was performed. All data were anonymised prior to analysis.

### Handling missing data

Two participants from the e-learning group did not complete the post-session questionnaire. To utilise all data, no list-wise deletion of data was used, as it was not required by the statistical test.

### Ethical considerations

Simulation training and e-learning are part of the ongoing training programmes participants take part in. The sessions did not deviate from what is expected of participants in their usual training. Participants were provided with an information sheet detailing the nature of the study, and they signed a consent form before participating in any teaching activities. The study was classed as a service evaluation according to the HRA decision-making tool. It was therefore registered as a service evaluation with the Welsh Ambulance Service NHS Trust.

## Results

### Participants

All 26 participants who volunteered for this study were eligible to participate and were divided into two equal groups. All 26 participants completed the pre-session questionnaire and all 13 participants from the telesimulation group completed the post-session questionnaire. Of the 13 participants in the e-learning group, 11 completed the post-session questionnaire.

### Demographics of participants

Table 1 provides a summary of the characteristics of the participants. Of the 26 participants, 22 (84.6%) were qualified paramedics, three (11.5%) were advanced paramedic practitioners and one (3.8%) was an emergency medical technician. No statistically significant characteristic differences were found between the telesimulation and e-learning group regarding occupation (p = 0.513), years qualified (p = 0.061), average participation in simulation training (p = 0.189) or average participation in e-learning training (p = 0.062).

**Table 1.** Demographics of participants.

Characteristic	Number (percentage) of participants		
Occupation	Telesimulation (N = 13)	E-learning (N = 13)	Total (N = 26)
Paramedic	11 (84.6%)	11 (84.6%)	22 (84.6%)
Advanced paramedic practitioner	2 (15.4%)	1 (7.7%)	3 (11.5%)
Emergency medical technician	0 (0.0%)	1 (7.7%)	1 (3.8%)
**Number of years qualified**			
<1	3 (23.1%)	6 (46.2%)	9 (34.6%)
1–5	2 (15.4%)	6 (46.2%)	8 (30.8%)
5–10	3 (23.1%)	1 (7.7%)	4 (15.4%)
10–15	2 (15.3%)	0 (0.0%)	2 (7.7%)
15–20	3 (23.1%)	0 (0.0%)	3 (11.5%)
>20	0 (0.0%)	0 (0.0%)	0 (0.0%)
**Average participation in simulation training**			
Weekly	0 (0.0%)	0 (0.0%)	0 (0%)
Monthly	1 (7.7%)	0 (0.0%)	1 (3.8%)
3–4 times a year	3 (23.0%)	6 (46.2%)	9 (34.6%)
Yearly	1 (7.7%)	4 (30.8%)	5 (19.2%)
Less than once a year	6 (46.2%)	2 (15.4%)	8 (30.8%)
Other: Never before	2 (15.4%)	1 (7.7%)	3 (11.5%)
**Average participation in e-learning training**			
Weekly	3 (23.1%)	1 (7.7%)	4 (15.4%)
Monthly	3 (23.1%)	10 (76.9%)	13 (50.0%)
3–4 times a year	3 (23.1%)	2 (15.4%)	5 (19.2%)
Yearly	2 (15.4%)	0 (0.0%)	2 (7.7%)
Less than once a year	2 (15.4%)	0 (0.0%)	2 (7.7%)
Other	0 (0.0%)	0 (0.0%)	0 (0.0%)

### Likert responses

**Figure 3:**
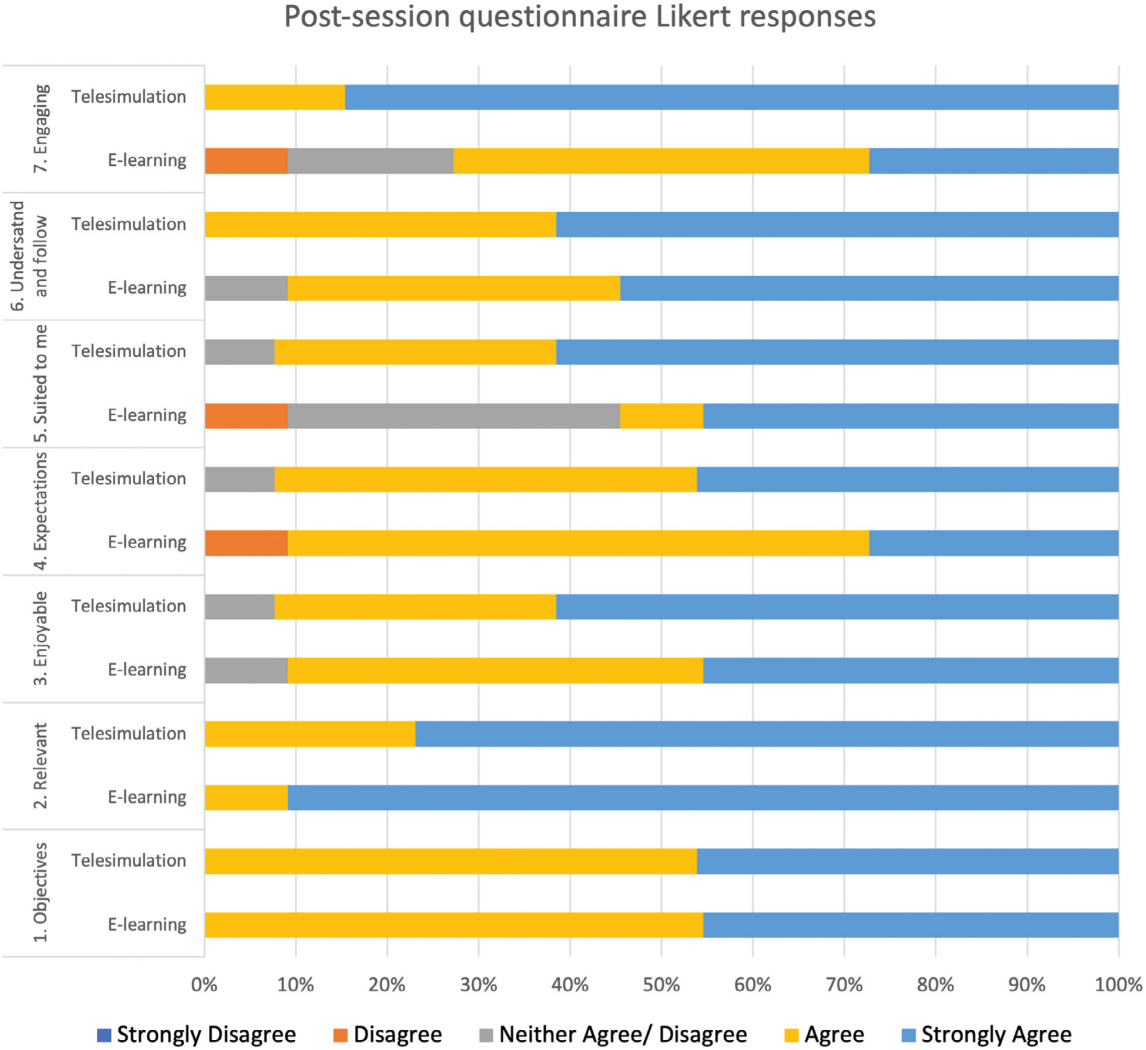
Figure 3. Participant responses to the post-session satisfaction questionnaire. The following statements were rated from ‘strongly agree’ to ‘strongly disagree’: (1) The objectives of the training were met; (2) This training was relevant to me; (3) This session was enjoyable; (4) This session met my expectations; (5) This style of teaching is suited to me; (6) I was able to understand and follow the session; (7) This session was engaging.

There was a positive response towards both teaching sessions (Figure 3). The participants confirmed that the training objectives were achieved, the teaching sessions were relevant and the sessions met their expectations. Participants reported both sessions as enjoyable and agreed the sessions were easy to follow and understand. However, there was a significant association between the groups and the response choice ‘This style of teaching is suited to me’. The telesimulation group was more likely than the e-learning group to agree that the session was suited to them χ^2^ (df = 1, N = 24) = 4.531, p = 0.033. Furthermore, the telesimulation group was significantly more likely to agree with the statement ‘This session was engaging’ χ^2^ (df = 1, N = 24) = 4.052, p = 0.044. There were no other significant associations between the groups and the other questionnaire items.

### Qualitative feedback

Table 2 lists the categories that emerged from the thematic analysis and includes key quotes provided by the respondents. The themes were grouped into advantages, disadvantages and improvements for telesimulation and e-learning. Categories emerged from these sections.

**Table 2.** Categories that emerged from the thematic analysis, including key quotes provided by the respondents.

Theme	Categories	Key quotes
1. Advantages of the telesimulation session	1.1 Enjoyment and engagement	‘Far more immersive than I would have anticipated, very realistic given the setup, enjoyed it more than I thought I would.’
	1.2. Learning	‘This method of learning worked really well, it was far better than just reading information and answering questions like in normal e-learning.’ ‘Heightened awareness for deflating chest in cardiac arrest.’ ‘Tiring and poor inspiratory effort will alert me to contact EMRTs [Wales Air Ambulance Service] for assistance if I was stuck somewhere remote.’
	1.3 Future training	‘I would certainly engage in further, similar simulation training.’
2. Disadvantages of and improvements for the telesimulation session	2.1 Introduction to team and allocation of roles	‘It may be beneficial to allocate roles, such as team leader, so everyone is able to “run” the scenario. I’m aware I have a tendency to take over otherwise!’
	2.2 Technical issues	‘The sound, there was a bit of feedback at times.’
	2.3 Familiarity with telesimulation	‘As this style of learning is new, I believe it will get easier and more intuitive for the learner over time.’
3. Advantages of the e-learning session	3.1 Learning	‘Good depth of knowledge and relevant to my role. The questions were difficult enough to promote thinking and keep me engaged.’ ‘The cardiac arrest section was information that I had long forgotten or never been taught – manual deflation and decompression – so thank you for that.’
	3.2 Enjoyment	‘I enjoyed the style of learning and the fact if we got an answer wrong, I could keep going until I got it right and your explanations why I got an answer incorrect were brilliantly explained.’
	3.3 Flow	‘Easy to follow and full of information and learning.’
4. Disadvantages of and improvements for the e-learning session	4.1 Content	‘More in-depth teaching of the anatomy and physiology.’ ‘Perhaps include post reading questions after every session which would be useful to consolidate and check learning.’
	4.2 Technical issues	‘Some of the slides with videos didn’t pop out and covered the diagram but that could of been due to me using an iPad rather than a laptop.’

## Discussion

Distance learning can resolve the dilemma of how to provide high-quality education where the number of participants is small and widely scattered (Davies et al., 2005); therefore, our aim was to evaluate the use of e-learning compared to telesimulation in the pre-hospital setting.

### Learning objectives

This study demonstrated the successful implementation of online learning around managing acute paediatric asthma. All participants agreed that the learning objectives of each session were met. Studies have identified challenges regarding the successful delivery of educational content through online learning (Astani et al., 2020; Jewer et al., 2019). Our study highlights the importance of focusing learning outcomes on what can be achieved through an online platform. They should also reflect the three key domains of learning identified by Bloom: cognition, comprehension and critical thinking (Krathwohl, 2002). Telesimulation and e-learning can assess learning on a variety of cognitive levels, which was demonstrated in our results. Telesimulation was viewed as a beneficial learning experience, with participants expressing high levels of satisfaction with this modality. The scenario drove learners towards higher levels of learning by requiring them to analyse and synthesise information. Although some participants were unfamiliar with this style of learning, we feel learners will become more confident through more exposure and will benefit greatly. Simulation-based education is a form of experiential learning; as described by Kolb, it is ‘the process whereby knowledge is created through the transformation of experience’ (Kolb, 1984). It can be argued that e-learning does not address this aspect, but solving a clinical scenario on an e-learning platform with clear learning outcomes can impart a similar experience for the learner.

### Engagement

Both the telesimulation session and e-learning module received positive feedback and were considered enjoyable. However, it appeared participants felt the telesimulation session was more engaging and the style of learning was more suited towards them. An important factor we considered was to ensure all participants were engaged throughout the telesimulation session and contributed towards the decision making. The facilitator’s role was designed to promote involvement and interaction by only responding to acts verbalised by participants. Patel et al. (2020) suggested small group sizes should be used during these sessions to improve engagement. Groups in our study only consisted of between two and four participants, and our results reflected their positive attitudes towards feeling engaged. The positive views towards engagement also support previous studies demonstrating telesimulation can be an enjoyable and engaging modality to deliver medical education to remote learners (Jewer et al., 2019; Mileder et al., 2021).

### Benefits to pre-hospital responders and their practice

Pre-hospital responders are scattered across regions and work various shift patterns, which can make attending training centres impractical. Remote learning can aid these responders with their development as clinicians by making education and training easily accessible. Both e-learning and telesimulation received positive feedback and have allowed a continuity of education flexibility. Participants provided positive feedback on their reaction to the teaching session (Level 1 of the Kirckpatrick Model (Heydari et al., 2019)) and also recall what they had successfully learnt on acute asthma management (Level 2). Practitioners also expressed how they will apply this knowledge to their practice and how their management of acute asthma will change. They also expressed how this training can improve patient outcomes. Future evaluation could confirm such applications (Level 3 and Level 4).

### Pedagogical choices

Clinical education and training for paramedic practitioners are usually classroom based and didactic (Nilsson et al., 2010). The theory and practice of managing ill paediatric patients is delivered using several pedagogical strategies like lecturing, demonstrating, prompting, question and answer, as well as intervening. Some, if not all, can be fulfilled by simulation-based education. Simulation can be cost- and resource-intensive; e-learning is, in comparison, low cost and does not exhaust resources. On the pedagogical aspect, it can fall short. We argue that in the circumstance of managing ill paediatric patients, the experience of paramedic practitioners during our e-learning session can be comparable to experiential learning, stimulating reflection and the desire to probe for knowledge.

### Limitations and challenges

#### Sample size

One limitation of this study is the small sample size, which is due to the voluntary nature of the study and the disruptions caused by the Covid-19 pandemic; it is therefore not a true representation of all pre-hospital responders. Also, a sample size calculation was not completed, which may influence some of the inferences made.

#### Technology

To mitigate technical errors, multiple dry runs of both the telesimulation and e-learning sessions were conducted to identify any key issues. However, some technical issues still occurred and were expressed in participant feedback.

One of the challenges with the set-up of the telesimulation scenario was ensuring good sound quality, minimising interference and microphone feedback. Some participants mentioned that microphone feedback caused some miscommunication. Furthermore, the platform used to deliver the educational content for our e-learning module, although it was practical to use, had some drawbacks. It disadvantaged some participants, as they were unable to view videos and pictures clearly due to layout and formatting issues when using a tablet device.

#### Fidelity

To improve the realism of the scenario, ideally the location would replicate the normal working environment inside an ambulance. However, due to the constraints of the technology and resources available, we were unable to achieve this. To overcome this, the simulation suite was designed to replicate the layout of the interior of an ambulance.

## Conclusion

Telesimulation and e-learning are effective modalities for delivering medical education for paramedic practitioners who work in geographically remote areas. Although this can be a reasonable alternative to face-to-face training, it still comes with challenges, such as online access, reliable connectivity and time for synchronous activity. E-learning does rely on learner engagement and lacks the immediate input from a facilitator; therefore, it can be difficult to impart a similar pedagogical experience. It can, however, apply some of the same strategies, such as prompting, didactic knowledge dissemination and encouraging critical thinking. Our study has shown that telesimulation using online telecommunication platforms and remote attendance of participants is effective for delivering clinical education. This can be a valuable resource for paramedicine education. We have endeavoured to evaluate the experience of telesimulation by comparing it to e-learning, a recognised modality that the practitioners are very familiar with. Further study is required to compare the experience of telesimulation with face-to-face experiential learning. There is also a need for evaluation of these different modalities on the organisational resources and impact on patient care.

## Acknowledgements

We would like to thank the Research and Development department at the Welsh Ambulance Service, the North Wales Clinical School and Dr Jason Walker for all their relentless support and hard work towards this study.

## Author contributions

CS, NR and SM were responsible for study conception and design. CS and SM carried out data collection and analysis and interpretation of results. CS and SM wrote the article; CS NR and SM were responsible for draft manuscript preparation. All authors reviewed the results and approved the final version of the manuscript. CS acts as the guarantor for this article.

## Conflict of interest

None declared.

## Ethics

Not required.

## Funding

This project was funded by Cardiff University School of Medicine. Two HD PTZ cameras, a microphone, CAE Learning Space™ and the Microsoft HoloLens glasses were provided by CAE (CAE, Montreal).
